# Supporting transitions in medical career pathways: the role of simulation-based education

**DOI:** 10.1186/s41077-016-0015-0

**Published:** 2016-06-03

**Authors:** Jennifer Cleland, Rona Patey, Ian Thomas, Kenneth Walker, Paul O’Connor, Stephanie Russ

**Affiliations:** 1 Institute of Education for Medical and Dental Sciences, University of Aberdeen, Room 1:132 Polwarth Building, Foresterhill,, AB25 2ZD UK; 2 Raigmore Hospital, NHS Highland, Inverness, UK; 3 School of Medicine, NUI Galway, Galway, UK

**Keywords:** Junior Doctor, Deliberate Practice, Boot Camp, Team Task, Repeated Practice

## Abstract

Transitions, or periods of change, in medical career pathways can be challenging episodes, requiring the transitioning clinician to take on new roles and responsibilities, adapt to new cultural dynamics, change behaviour patterns, and successfully manage uncertainty. These intensive learning periods present risks to patient safety. Simulation-based education (SBE) is a pedagogic approach that allows clinicians to practise their technical and non-technical skills in a safe environment to increase preparedness for practice. In this commentary, we present the potential uses, strengths, and limitations of SBE for supporting transitions across medical career pathways, discussing educational utility, outcome and process evaluation, and cost and value, and introduce a new perspective on considering the gains from SBE. We provide case-study examples of the application of SBE to illustrate these points and stimulate discussion.

## Background

Transitions are inherent in medical education, training, and working life. However, evidence from healthcare and other literature indicates that transitions can be challenging for medical students and doctors, who report feeling underprepared in terms of technical and non-technical skills and who report high levels of associated stress [1–4]. Calls have been made for further formalised training aligned to support doctors in tackling the specific challenges experienced during educational transitions [5]. In this paper, we discuss the potential utility of simulation-based education (SBE) as a mechanism to support transitions in medical careers. We provide some examples of how this is already happening and suggest ways to expand the use of SBE in terms of preparedness for clinical practice in the broadest sense. Whilst identifying concrete specific outcomes of SBE for this purpose is not the focus of this paper, we situate our argument in the wider literature on how formal education and practice-based experiences contribute to the development of medical capacities and dispositions [6] and suggest ways to ensure maximum gain from SBE.

## Simulation-based education

First, it is important to be clear what we mean by SBE. Simulation is a means of allowing deliberate hands-on practice of clinical skills and behaviours prior to, and alongside, entry into clinical environments. The aim of SBE is to develop safe clinicians by creating alternative situations and environments in which to learn skills and behaviours. SBE encompasses a breadth of approaches, from low-cost bench simulators to high-fidelity manikins, from simulated patients for learning communication skills to complex ward simulations and haptics.

Simulation is required in medical education for a number of reasons. The natural method of teaching clinicians advocated by Osler (1903)—unstructured clinical experience—was shown to be educationally ineffective [7]. Therefore, the focus of medical education and training shifted to a competency- or outcome-based model of teaching and learning where objectives and outcomes, assessment and feedback, and practice and supervision became the norm [8]. Concurrently, reduced availability of patients for teaching and learning medicine, due to changes in healthcare delivery [9], as well as increased emphasis on protecting patients from unnecessary harm [10], placed limits on the nature of patient contact, particularly for relatively inexperienced learners. Last but not least, in many countries, including Europe and to a lesser extent the USA, hours of training have now been strictly controlled by working time legislation which also led to increased interest in alternative pedagogic paradigms. SBE addresses all of these issues—decreasing reliance on training on real patients, allowing for instant feedback for correction of errors and for directing learning, optimising use of valuable clinical time, enhancing the transfer of theoretical knowledge into the clinical context, and ensuring learners are competent before exposure to real patients [11–13]. SBE focuses on the needs of the learner—it allows for the deconstruction of clinical work patterns to focus on the mastery of a particular skill or combination of skills of interest, it can be calibrated to meet the needs and level of the individuals or teams involved, and it can be optimised in terms of timing of delivery to support skill development in a preparatory fashion [13]. Additionally, given that individuals find it difficult to reliably self-assess their level of preparedness—their strengths and weaknesses [14]—SBE incorporating rigorous, objective, and relevant measures of performance can contribute stronger predictive data regarding readiness for a role and help to identify areas requiring focused educational attention and self-directed learning prior to making a transition. Research has indicated a positive relationship between SBE and learning outcomes including the development of technical and non-technical skills, confidence, and, critically, patient outcomes [15–18]. Indeed, a number of recent publications have identified that SBE can have a measurable, direct effect on a range of patient and public health outcomes including ICU infection rates, lower childbirth complications, and reduced post-operative complications and overnight stay [19–21].

## How can SBE support transitions in medical education?

It is useful to think of the complexity of transitions before answering this question. As defined by Kilminster et al., the term transition refers to the process of change or movement between one state of work and another [22]. At the undergraduate level, transitions start with entry into medical school and then involve moving from non-clinical to clinical environments and rotating through different medical specialties, culminating for many in the transition from medical school to working as a junior doctor. Following graduation, junior doctors rotate from unit to unit, place to place, speciality to speciality, and then sub-specialty to sub-specialty, before moving on from being a senior trainee or resident to their first fully trained post. Each transition presents an intensive learning period, requiring that the individual adapt to new environments, with their values, norms, and beliefs [23], manage uncertainty, master unfamiliar equipment or technology, work with new colleagues, and perhaps take on new roles/responsibilities and/or work with unfamiliar patient groups. Given the potential “breadth” of unfamiliarity associated with the changing working environment, it is perhaps understandable that transition points present risks for patient safety [24] and may stifle progress in skill acquisition [25, 26].

The focus of the majority of the research on transitions has been that of the move from medical school to junior doctor or internship (Foundation year 1 in the UK), where the learning curve is steep and the challenges facing new doctors are well defined and relatively well understood. During this transition, the emphasis shifts from learning to balancing education with performing a role in the workplace. Research shows that new doctors often feel they lack the skills and competences for work upon graduation [2]. Studies from the UK context suggest that there are particular areas in which senior medical students or new doctors feel unprepared, such as clinical reasoning and making a diagnosis, diagnosing and managing acute medical emergencies, and prescribing, as well as competencies associated with non-technical skills such as communicating effectively in a multidisciplinary team, speaking up, prioritising patients, handover, and breaking bad news [1, 27–29]. (Note that this feeling of being thrown in at the deep end is not unique to medical graduates: the messages from the literature on the transition from student nurse to staff nurse are very similar) [30]. There is a paucity of research around transitions later in medical training where, arguably, the role shifts are less dramatic, but what evidence there is suggests that the issues are broadly the same. What we know is that those transitioning from Foundation doctor to specialist trainee (intern to resident) often report a heavy focus on service delivery to the detriment of having time to learn and develop new skills, to pursue sub-specialty interests and to gain exposure to the responsibility necessary for progressing in their roles [31]. Similarly, doctors transitioning from specialist trainee (senior resident) to consultant frequently recognise that they are deficient in several necessary non-clinical skills, e.g. supervision, handling complaints, decision-making, delegation, managing conflict, and providing feedback [32]. Although, as yet, there has been relatively little research on the effects of these later career transitions on doctors’ performance and patient safety, it would seem prudent at this point in time to consider all transitions in medical education and training as challenging and with the potential to lead to harm if poorly managed by the individual and the system.

SBE can aid transitions by allowing medical educators to create the conditions in which learners can undergo the practice to acquire and maintain essential (pre-determined) skills, behaviours, and expertise [33]. Learners can rehearse specific skills and procedures and practise broader tasks such as managing competing demands in acute settings, in artificially created environments which are designed to be authentic and to facilitate acquisition of expertise by individuals and teams, via practice, assessment, and feedback [34]. By doing so, learners are better prepared for clinical practice and hence may be able to manage transitions more effectively. How does this work? Increased knowledge and skill obtained through SBE allows necessary information to be accumulated and stored in long-term memory, and drawn on as required, freeing working memory to focus on other aspects of the task in question. To borrow an example from Leppink et al. [35], a novice reviewing an x-ray for the first time may see a mass of different elements all of which need to be processed to make sense of the x-ray. A more experienced learner, who has learned about x-rays and who has a preliminary cognitive schema of what to expect (in terms of physiology, anatomy, and imaging), can make sense of the x-ray more easily. This leaves him or her more cognitive resources to process other unknown aspects of the task. The same applies to practising any other skill, for example non-technical skills such as patient prioritisation or task delegation—once these skills have been rehearsed and incorporated into cognitive schema in the long-term memory store, they will become more automatic and capacity to process additional information simultaneously will increase. Thus, SBE prepares the individual to manage challenging and new situations by supporting them to learn parts of the puzzle in advance (so information is in storage to draw on as required), thus freeing up working memory to focus on what is new, novel, or unexpected [36]. In short, SBE draws on years of research into deliberate practice [8, 35] and cognitive load in a number of high-risk areas—not just medical education but also aviation, oil and gas, and energy [37, 38]—to create safe learning conditions for learners, to ensure safety in real-life situations.

**Fig. 1:**
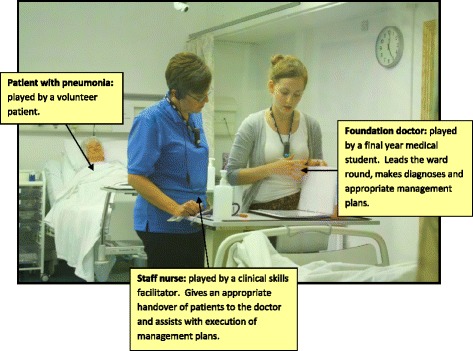
A simulated ward round experience: bridging the gap between undergraduate and postgraduate years

Whilst the theoretical basis of SBE is well-recognised and researched, there has been less of a focus on using SBE effectively to support transitions in the medical career pathway. To effectively use SBE to support transitions requires a number of considerations which draw on the wider literature on deliberate practice. First, the simulated scenarios or tasks must be linked to well-designed learning objectives which are appropriately aligned with the learner’s stage of training and to areas known to be problematic at that transition point [7, 11, 39]. Given, for example, most UK medical students feel adequately trained in terms of basic medical knowledge, history taking, and certain clinical skills, but less confident in other areas, including non-technical skills, such as prioritisation and teamwork [29], then SBE to support the transition between medical school and internship could usefully focus on the latter areas. To give an example of using SBE to support the development of non-technical skills, a number of years ago, we identified that newly qualified doctors struggled with seeking help from senior staff in out-of-hours situations, particularly where communication was by telephone. We developed a simple simulation-based session which involved realistic scenarios, a phone, clinical staff taking the calls, feedback from those taking the calls and faculty (observing the student making the call), and “handy hints”. Our data indicates that the block of teaching of which this is part (the “Professional Practice Block”) has been effective in graduating doctors who are more prepared for practice [40]. Second, for SBE to be effective, it should be integrated into the curriculum in a way that promotes transfer of the skills learnt to clinical practice. For example, it should be initiated at the appropriate educational moment/s, it should include processes for reinforcing learning (including immediate and informative feedback with a focus on areas of weakness), there should be opportunities to amend behaviours (i.e. time for reflection and consideration of current strategies and repeat sessions to allow learning to be put into practice), and, ideally, it should be possible to track performance gains (or losses) using rigorous and objective performance measures [11, 41, 42]. An example of SBE which was grounded in observable difficulties at the time of transition from medical school and included many of these essential features of deliberate practice was recently published by Thomas and colleagues (Table 1, Fig. 1) [43].

**Table 1:** Simulated ward round to support the transition from medical student to junior doctor

Junior doctors are particularly susceptible to error-making within stressful environments. The ward is an excellent example—for it is endemic in distraction [64]. Through overwhelming cognitive load, distraction impairs clinical reasoning [65] and contributes to prescribing error [66]. Despite this, medical graduates receive little training in how to cope with hectic work environments and it is perhaps unsurprising that the early years of a medical career are the most error prone [67]. However, the literature suggests that practice with distraction and interruption can dampen their adverse consequences [68]. In response, Thomas and colleagues investigated whether a simulated ward exercise could improve medical student management of distractions to reduce error-making.	
A high-fidelity simulated ward round experience was developed. Students played the part of a junior doctor leading the round and completed a number of error-prone tasks, from patient diagnosis to prescribing. At time-critical points, common distractions were deployed (for example, the doctor’s pager being set off or having to deal with a disgruntled relative) (Fig 1).	
A non-randomised controlled study was undertaken with 28 final-year medical students. All students participated in a baseline ward round. Fourteen students formed an intervention group and received immediate feedback on their handling of distractions. The 14 students in a control group received no such feedback. After a lag-time of 1 month, students participated in a post-intervention ward round of comparable rigour. Changes in medical error-making and distractor management between simulations were evaluated.	
Baseline error rates were high, with 72 and 76 errors witnessed in the intervention and control groups, respectively. Many errors were life threatening and included prescribing patient-allergic antibiotics, inappropriate thrombolysis, and medication overdoses. Similarly, at baseline, distractions were poorly managed in both groups.	
All forms of simulation training resulted in error-reduction post-intervention. In the control group, the total number of errors fell to 44, representing a 42.11 % reduction (*p* value = 0.0003). In the intervention group, the total number of errors fell to 17, representing a 76.39 % reduction (*p* value <0.0001). The management of distractions only improved significantly in the intervention group.	
Students highly valued the simulations [69], which were deemed high fidelity and built confidence.	
“I really hope this is a method of education that catches on because I feel it has been one of my most valuable learning experiences in 5th year so far.”	
The research shows that students are not inherently equipped with the skills to manage distractions in order to mitigate error. However, practice with distractions minimises its adverse consequences and targeted feedback is key in achieving the greatest educational utility.

**Fig. 2:**
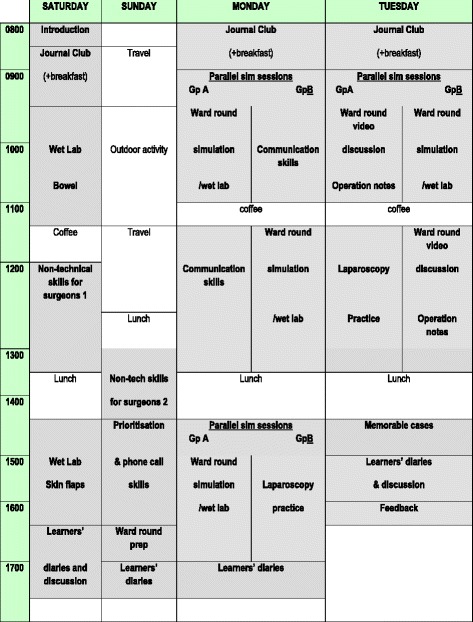
Scottish Surgical Boot Camp programme, 2015

Fewer SBE examples exist to support more senior transitions in a doctor’s career. This is reflective of the relative paucity of research focused on understanding the challenges faced during these later transitions and is mirrored by the fact that whilst the competencies required by junior doctors have generally been outlined by national training bodies, this is not typical for more senior transitions in medical careers. Nevertheless, some promising work is emerging here. One relatively new approach to SBE is the “Boot Camp”. Boot Camps are themed on the principles of a military Boot Camp—intensive, focused training using experiential learning and hands-on practice to learn new skills and knowledge in a safe environment. Several US Boot Camps, designed to support interns transitioning into residency programmes, and drawing heavily on SBE for technical skill development, report improvements in interns’ confidence levels and procedural skill acquisition following repeated exposures to clinical scenarios in a simulated setting [44–46]. A further example of a Boot Camp to support the transition from junior doctor into a surgical training programme in the UK (Fig. 2), one which includes a novel focus on developing core non-technical skills through SBE, is provided in Table 2 [47].

**Table 2:** Scottish Surgical Boot Camp

In designing the Scottish Surgical Boot Camp (SSBC), the surgical training faculty in Inverness, Scotland, set out to define and include skills, attitudes, and even values that seemed essential for a safe and “flying” start to surgical training. The content was derived from their observations as trainers of where surgeons (especially trainees) tend to struggle and of which skills had previously been learned “the long way” by apprenticeship or prolonged clinical exposure, or sometimes never learned at all, and which now could be taught early on using a new paradigm of training. Hence, alongside technical skills such as bowel anastomosis and laparoscopic instrument handling, the programme includes sessions devoted to non-technical skills in complex real-life settings, e.g. the leading of a simulated ward round in the face of distractions and the handling of difficult written or spoken communication scenarios. Also included are anecdotal lessons in resilience.	
First piloted in 2011, the SSBC was adopted in 2014 by the two Core Surgical Training programmes in Scotland as their introductory course for new start trainees, endorsed by two surgical Royal Colleges (Edinburgh and Glasgow) and fees are subsidised by the NHS Education for Scotland, the body which oversees training for all doctors and healthcare professionals.	
The current iteration of the programme is shown in Fig. 2. Most sessions include SBE, and it is not difficult to see from the programme how as a whole it mimics the structure of a “surgical day” and “surgical week”. Also built in is an adherence to Issenberg’s highly evidence-based 10 conditions for effective simulation-based learning [70]. For example, skills are practised in a variety of clinical settings, in valid and controlled simulations, with immediate and individualised feedback.	
The technical tasks taught and practised using pig tissue in the “wet labs” are limited to two defined, useful tasks not easily accessible to the new start trainee in real clinical practice, which require discipline and repetition and in which the learner rapidly feels the benefit of repetition. These are small bowel anastomosis, skin flaps, and/or tendon repair. The non-technical skills are taught using the well-established taxonomy “Non-Technical Skills for Surgeons” [71] (NOTSS) and using varying simulated phone call scenarios and a simulated ward round with detailed individual feedback on the core NOTSS behavioural constructs (situation awareness, decision-making, communication and teamwork, and leadership).	
Educational theory has been used to understand the complex processes of the Boot Camp by way of a qualitative study [72].	

Projecting further ahead still, to the transition from specialty training to career grade positions (attending clinicians in the USA and consultant posts in the UK and Europe), it was not possible to identify any SBE approaches designed to support the development of the new skills and behaviours necessary for mastery of these roles. Since the emerging literature is starting to highlight a need for more support for certain non-technical elements of these positions (e.g. supervision, delegation, influencing culture) [32], it may be time to define and specify the precise learning objectives that can be addressed usefully via deliberate practice and to plan SBE interventions that can support preparedness at these higher stages.

## What other considerations are important when planning SBE to support transitions?

First, in relation to feedback, it is critical to use trained faculty, who are skilled in giving immediate, informative feedback and engaging participants not just with SBE generally but also with the feedback component of SBE. More contemporary theories of feedback stress the importance of the learner in the feedback process, conceptualising feedback as a dialogic process, where effective feedback depends on learner engagement and activation of the internal regulatory processes required for goal-directed learning [48]. To do so sets expectations for the role of both faculty and learners in the feedback process, which may differ from their experiences of feedback to date, and hence should be explicitly considered in the training and preparation components of any SBE. Associated with this, and discussed earlier, SBE must be matched with performance standards, educational objective(s), and opportunities for repeated practice (with feedback) in order to reach these standards. Moreover, it is essential to set up SBE to recognise that different people require different amounts of practice to accomplish mastery of predefined educational objectives [49]. In other words, one learner might master the outcomes/objectives associated with a simulated anastomosis activity with related ease depending on their prior learning, hand-eye coordination, etc., whereas another learner with different skills, knowledge, experience, and/or attitudes towards learning may need repeated practice.

Clearly, as stated above, SBE outcomes need to be defined in advance. When evaluating SBE, it is very important to go beyond acceptability (e.g. “8 out of 10 students reported that they enjoyed the [SBE]”) and move into outcomes based on measurable change in skill acquisition, whether the objectives are technical or non-technical skills [17, 18], all the way to translation into practice—“from VR-to-OR” [50]—and better outcomes for patients [19–21]. Clearly, it is much easier to collect evaluation data immediately after an SBE intervention than it is to follow up learners when they are out in clinical practice, but it is essential to carefully plan long-term follow-up as otherwise it will be hard to justify the value of SBE to education providers and funders. One study assessed the impact of SBE on performance in the clinical environment in an Irish teaching hospital. SBE on ordering blood products was delivered to newly graduated medical students as part of a Boot Camp course prior to working as a junior doctor. The training was found to reduce the risk of a rejected sample by 65 % as compared with junior doctors who did not receive the training. Moreover, the risk of a rejected sample for trained interns was 45 % lower than for much more experienced doctors. The untrained interns required more than 2 months of clinical experience to reach an error rate that was not significantly different from that of the trained interns [51].

In addition to high-quality outcome evaluation, Moore et al. [52] discuss a number of benefits of complementing this with process evaluations (i.e. understanding the functioning of an intervention, e.g. How was it implemented?, What are the mechanisms of effect?) in relation to complex interventions (and we believe the educational interventions, including SBE, can be considered as complex interventions) [53]. Process evaluations provide the benefit of being able to inform the educators (e.g. was the simulated intervention delivered as intended, does it need to be redesigned in some way) and/or identify aspects of context which acted as barriers to the new learning being translated into clinical practice.

Value is usually related to cost [54]. Medical schools and medical training providers need to answer to governments, regulators, funders, and the public in terms of whether what they are delivering is fit for purpose. “Fit for purpose” can be considered from a number of perspectives. For example, are we producing the right doctors in terms of knowledge, skills, attitudes, and behaviours to meet the health needs of the communities they serve? Are we delivering these outcomes not just to a high standard but in a fiscally responsible way—can we justify a high-cost simulation over an apparently low-cost clinical experience? What are the gains from SBE that would be unobtainable, or unsafe, in more traditional models of teaching and learning? There is a need to develop an evidence base for SBE which encompasses “fitness for purpose” both educationally and fiscally. A high-cost, low-educational value SBE is the worst of all possible outcomes. A low-cost, low-value SBE will not meet anyone’s needs in the long term, whereas a high-cost, high-value SBE would probably be acceptable. Thomas and colleagues [55] calculated the cost of their simulated ward round and realised that the high cost limited the feasibility of the simulation as it was originally designed. By identifying the main cost components, they were able to develop and evaluate a slightly different approach (e.g. group feedback rather than individual feedback). Costs were significantly reduced, but the positive response from participants was maintained. As these are recent studies, it is not yet known if changing from individual to group feedback impacts differently on more distal outcomes such as clinical care practices. However, paying explicit attention to the cost of their SBE allowed Thomas and colleagues to consider other models of educational delivery without threatening the quality of their product.

Our next consideration is that of fidelity in the broadest sense. Much SBE has focused on individual skill development. However, healthcare is usually a team effort and many of the problems noted in transitions are about team skills, e.g. communication with other members of the multidisciplinary team, supervision, and speaking up across professional hierarchies [28–30]. This means it is essential both to develop SBE team tasks and to develop outcomes that go beyond individual gains to team outcomes. These group objectives might be “hard” outcomes such as systemic improvements in team performance (e.g. fewer errors, cost savings, more efficient patient throughput), but it is also important to assess softer, process-, and team-related outcomes, including the quality of inter-disciplinary teamwork in a global sense and including specific team tasks such as communication during handovers, transfer of leadership, and task-based coordination [56]. Through the observation of teams working together in realistic scenarios and reflecting on performance through debriefing (potentially facilitated by the use of video), SBE might also help to explore more complex team-based dynamics such as social hierarchy, diversity, and divisions which can be difficult to pick up in more traditional classroom-based approaches to inter-professional education [57]. In this sense, SBE might also be tailored to target any specific team-based issues ongoing at a local level [58].

Finally, whilst it is absolutely critical to know what works in SBE and understand how it does so in terms of individual, cognitive, and acquisitive learning, limiting the focus of research to outcome and effectiveness studies means understanding of SBE will remain limited. There has been, to date, no acknowledgement in the literature that much SBE is inherently a social activity, bringing together groups of learners and faculty, away from the everyday clinical environment, sometimes in residential situations. By not recognising this explicitly, we have no understanding of how the relationships between faculty, participants, and activities during SBE influence learning [59] or of the nature or influence of the hidden curriculum [60]. Nor do we know about the influence of the particular cultural contexts, for example, of the wider socio-cultural, institutional, and historical setting and complexities of clinical training [61], in which SBE is situated. The need to extend the range of approaches to researching this field is real because, if SBE is based on limited models of learning, it risks inadequately preparing learners for practice. Moreover, where theoretical frameworks are lacking, explanations of the simulation phenomena that can be elaborated and refined in future research may not be forthcoming. Indeed, recently, some researchers have called for the reconceptualising of simulation education generally, to draw on contemporary practice and to consider questions of learning in complex healthcare systems [62, 63].

## Conclusions

Increasing doctors’ preparedness to perform the skills and behaviours required to fulfil the responsibilities of any new role is important for patient safety, service efficiency, and individual psychological well-being. Whilst true mastery of a role cannot be achieved until one is immersed within the workplace itself [6], the literature indicates that we can go some way to preparing individuals for the technical and non-technical elements of any new role, and indeed the associated psychological challenges, through the judicious and imaginative use of SBE. In this paper, we have provided an overview of some of the key factors associated with planning and evaluating SBE for transitions. We have also highlighted a number of areas for future research in SBE to support medical career transitions. These include the development of understanding around the practical factors to be considered when designing SBE, ranging from the delivery of feedback and the incorporation of longer term outcome measures to analysis of the cost-effectiveness of the approach being undertaken, as well as the socio-cultural influences on learning in simulated settings. We urge those working in SBE research to consider how best to identify and evaluate concrete specific outcomes of SBE for this purpose. There remains the need for further investigation into the use of SBE to support the transition from medical student to junior doctor, but we urge those working in this area to not neglect examining the use of SBE to support later medical career transitions where “learners” are working with less supervision and increasing responsibility yet where (largely non-technical) issues pertinent to patient safety remain apparent.
